# Proteomic analysis of serum in a population‐based cohort did not reveal a biomarker for Modic changes

**DOI:** 10.1002/jsp2.1337

**Published:** 2024-07-15

**Authors:** Friederike Schulze, Juhani Määttä, Sybille Grad, Irina Heggli, Florian Brunner, Mazda Farshad, Oliver Distler, Jaro Karppinen, Jeffrey Lotz, Stefan Dudli

**Affiliations:** ^1^ Center of Experimental Rheumatology, Department of Rheumatology University Hospital Zurich, University of Zurich Zurich Switzerland; ^2^ Department of Physical Medicine and Rheumatology Balgrist University Hospital, Balgrist Campus, University of Zurich Zurich Switzerland; ^3^ Research Unit of Health Sciences and Technology University of Oulu Oulu Finland; ^4^ AO Research Institute Davos Davos Switzerland; ^5^ Department of Orthopedics Balgrist University Hospital Zurich Switzerland; ^6^ Rehabilitation Services of South Karelia Social and Health Care District Lappeenranta Finland; ^7^ Department of Orthopaedic Surgery University of California San Francisco San Francsisco California USA

**Keywords:** degenerative disc disease, low back pain, Modic changes, northern Finland birth cohort, proteomics, serum‐biomarker

## Abstract

**Introduction:**

Modic changes (MC) are bone marrow lesions of vertebral bones, which can be detected with magnetic resonance imaging (MRI) adjacent to degenerated intervertebral discs. Defined by their appearance on T1 and T2 weighted images, there are three interconvertible types: MC1, MC2, and MC3. The inter‐observer variability of the MRI diagnosis is high, therefore a diagnostic serum biomarker complementing the MRI to facilitate diagnosis and follow‐up would be of great value.

**Methods:**

We used a highly sensitive and reproducible proteomics approach: DIA/SWATH‐MS to find serum biomarkers in a subset of the Northern Finland Birth Cohort 1966. Separately, we measured a panel of factors involved in inflammation and angiogenesis to confirm some potential biomarkers published before with an ELISA‐based method called V‐Plex.

**Results:**

We found neither an association between the serum concentrations of the proteins detected with DIA/SWATH‐MS with the presence of MC, nor a correlation with the size of the MC lesions. We did not find any association between the factors measured with the V‐Plex and the presence of MC or their size.

**Conclusion:**

Altogether, our study suggests that a robust and generally usable biomarker to facilitate the diagnosis of MC cannot readily be found in serum.

## Graphical abstract

A serum proteomic analysis of a subset of the Northern Finland Birth Cohort 1966 with DIA/SWATH‐MS revealed no biomarker for Modic changes. Further, a measurement of a panel of inflammatory markers did not confirm previously published biomarkers for Modic changes.

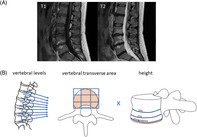

## 1 INTRODUCTION

Modic changes (MC) are inflammatory bone marrow lesions adjacent to degenerative vertebral discs.^1^ They are one potential cause for chronic low back pain (cLBP), which is a major global health burden and one of the leading causes of disability.^2^, ^3^ MC can come as MC1: hypointense on T1, hyperintense on T2 weighted images, MC2: hyperintense on both T1 and T2 weighted images, and MC3: hypointense on both T1 and T2 weighted images. Especially MC1 is associated with cLPB, and the larger the lesion, the stronger is the association with pain.^4^ One type of MC can convert to another and there are lesions with more than one type of MC (so‐called mixed MC type, MC1/2 or MC2/3). The only way to diagnose MC to date is by magnet resonance imaging (MRI) with a high inter‐observer variability and often a binary present/absent diagnosis without further detail about the type or the extent. The only way to follow up the development of MC is to repeat MRI.

Recently, there have been attempts to find serum biomarkers for the presence of MC. This could add a cost‐effective way to support MRI diagnostics and to follow up the development of the MC in patients without repeat MRI.

In a study with 12 patients hospitalized for cLBP, in patients with MC1, the serum concentration of high‐sensitive C‐reactive protein (hs‐CRP) was higher than in patients with MC2 or without MC (noMC).^5^ Karppinen et al. tested proteins involved in inflammation, vascularization, and bone turnover in 40 patients with cLBP and MC and 40 matched controls without cLBP. They found 15 proteins, which were reduced in serum of patients compared to controls, and two proteins, which were increased. Hs‐CRP was not significantly different.^6^ Gjefsen et al. measured 40 cytokines in 46 patients with MC1 and 37 patients with MC2 with cLBP, respectively, and 50 controls without cLBP. They found six cytokines to be increased in MC1 and five to be increased in MC2. The most important one was macrophage migration inhibitory factor (MIF).^7^ Aboushaala et al. tested 81 potential biomarkers in a cohort of 31 patients undergoing spinal fusion or microdisectomy surgery with either MC (not further specified which type) or noMC. They found, among others, MIF to be decreased in MC.^8^

These studies^6^, ^7^, ^8^ did not measure exactly the same markers, but for the markers measured in both studies, the data are conflicting. We found markers for collagen degradation (C4M, PRO‐C4, and PRO‐C3) to be elevated in the serum of 21 patients with any MC compared to 33 with noMC, and in 10 patients with cLBP and MC1 and 11 patients with MC2 compared to 19 patients with cLBP and noMC.^9^ Boisson et al. found no difference in serum concentrations of the markers in their panel of inflammatory markers, markers of redox status, and cartilage degradation in 34 patients with cLBP with and without MC1,^10^ partly conflicting with the studies mentioned above. In a large cohort study, Li et al. found the diameter of VLDL to be decreased in participants with MC.^11^ In summary, none of the significant results of any study has ever been reproduced.

The limitations of these studies (with the exception of Li et al.) are, that various methods were used to measure preselected factors on a preselected population. This produced various results for which it is not clear if they can be transferred to a general population. We summarized the significant results in Table 2 and all results in Table S2.

We aimed to find serum biomarkers to complement the MRI diagnostics to better detect MC and possibly be able to follow up on the development of the MC without repeat MRI. For this, we analyzed prospectively collected data and samples of the Northern Finland Birth Cohort 1966 (NFBC1966) with data independent acquisition sequential window acquisition of all theoretical mass spectra (DIA/SWATH) mass spectrometry (MS). DIA/SWATH is a cutting‐edge unbiased, reproducible, and sensitive MS approach with unprecedented depth and precision.^12^ For a better comparison with previous studies, we also measured a panel of cytokines and chemokines with an ELISA‐based method.

## 2 MATERIALS AND METHODS

### 2.1 Subjects, data, and serum collection

The present study is a follow‐up study of the NFBC1966 and was approved by the regional ethical committee of the Northern Ostrobothnia Hospital District, Finland, as well as the ethics commission of the canton of Zurich, Switzerland. The NFBC1966 is a large population‐based cohort, initially comprising over 95% of all children born in Northern Finland in 1966.^13^, ^14^ After the latest follow‐up in 2012 (mean age = 46.3), participants living near Oulu (*n* = 1988) were additionally asked to undergo a spinal MRI, which *n* = 1534 participants did.^3^ Of those, 326 participants were selected for participation in this study: 158 patients with MC, half of whom had cLBP, were selected for the highest expected effect size based on the image data (large MC/many MC) and matched with 168 participants with noMC, half of whom had cLBP.

Serum sampling and questionnaire data collection were done as specified in the NFBC1966 study protocol, which is available on the NFBC1966 website. In brief, questionnaires (among others on pain, smoking status, etc.) were sent to all participants with the invitation to the 2012 visit of the study, to be filled either online or, if requested by the participant, by paper. Blood samples were taken on the day of the 2012 visit after a 12 h fasting period. Blood was drawn into serum tubes (BD vacutainer, REF 367896, Nümbrecht, Germany) and centrifuged for 11 min at 2200 g within 30–60 min after collection. Thereafter, the supernatant, that is, the serum, was frozen. All shipment of the samples were on dry ice. Storage was on −80°C. Every participant of the present follow‐up study gave her or his consent for data and biomaterial usage.

### 2.2 MRI, MC classification, and MC load

The MRI scans have been reported before.^3^ In brief, T1 and T2 weighted sagittal‐plane images and T2 weighted axial‐plane images were obtained using a 1.5 Tesla MRI scanner (Signa HDxt; General Electric, Milwaukee, WI) using the same imaging protocol for all subjects.

**FIGURE 1:**
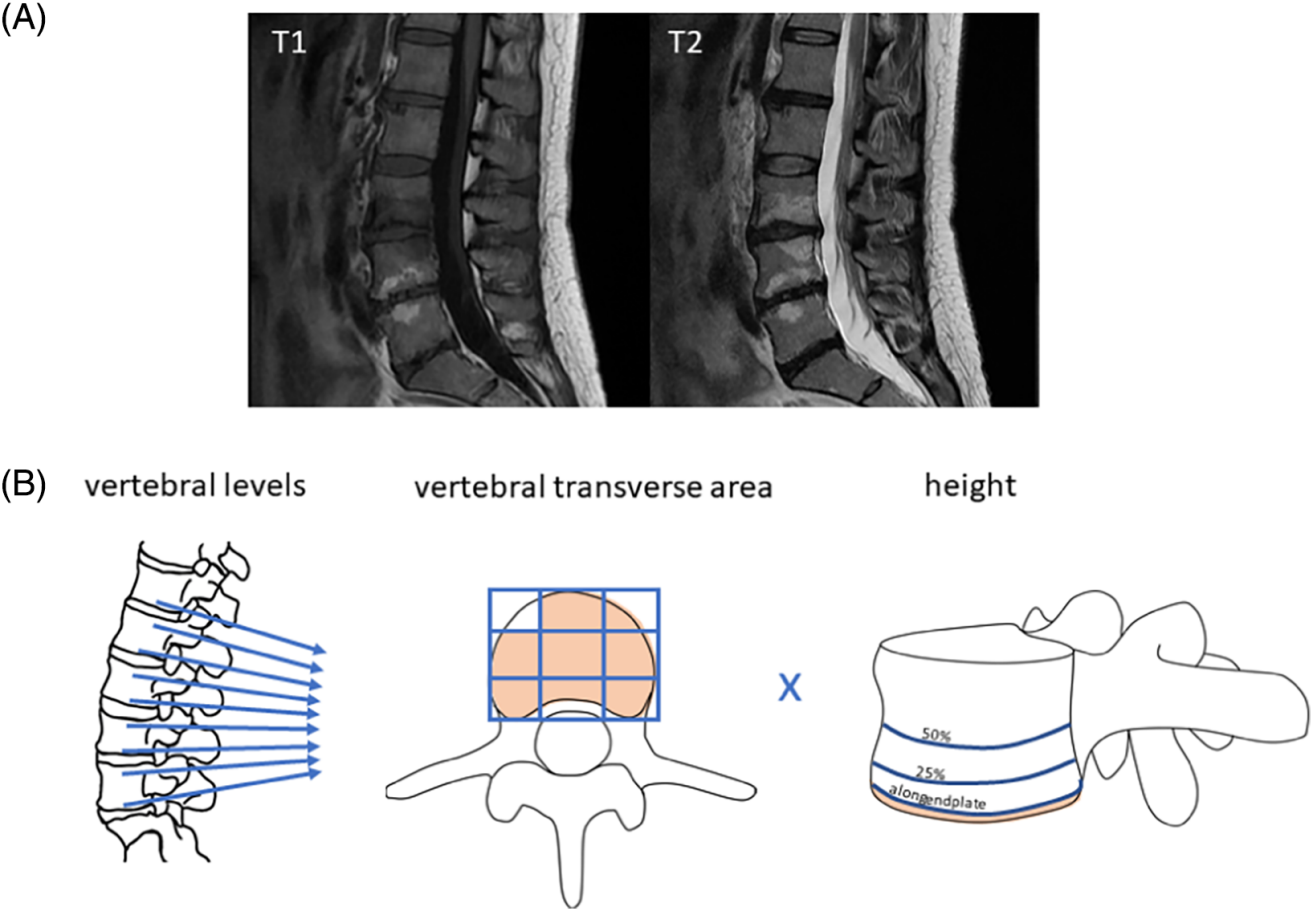
(A) Representative MR images of a subject with four partly mixed MC: predominantly MC1 at the lower endplate of L3, MC1 at the top endplate of L4, predominantly MC2 at the lower endplate of L4 and the top endplate of L5. (B) Calculation of MC load: We considered MC at the rostral and caudal endplate adjacent to the five intervertebral discs L1‐S1. The transversal area of the MC was measured as ninths of the transversal area of the endplate, here, for example, 8. The height of the MC was given in 4 groups: 1 = “along the endplate”, 2 = “>25%,” 3 = “25%–50%” and 4 = “<50% of the height of the vertebral body,” here, for example, 1. The MC‐load was calculated as Σ (surface (MC) [1/9 parts of vertebral endplate surface] × height (MC).^1^, ^2^, ^3^, ^4^).

Two independent researchers analyzed and classified MC on the T1‐ and T2‐weighted images, and, in case of discrepancy agreed on a common rating. They rated the type, location, transverse area, and height of each MC in the lumbar spine as published previously.^3^ Transverse area of MC lesions was given as ninth parts of the vertebral endplate surface of a fictitious 3 × 3 grid. Height of MC lesions was given in four categories: (1) “along the endplate,” (2) “<25% of the height of the vertebral body,” (3) “25%–50% of the height of the vertebral body,” and (4) “>50% of the height of the vertebral body.”^15^ From this, we calculated the extent of each MC type for each participant, which we called “MC load” (Figure 1). In case of mixed MC type, we split the MC load 1:1 between the types. Disc degeneration was assessed on the same images and reported level‐wise as Pfirrmann grade (1–5).^16^ As a measure for the disc degeneration of lumbar spine, we calculated a Pfirrmann score of the lumbar spine as sum of the Pfirrmann grades of L1/L2–L5/S1.^8^ The score can have values between 5 and 25. We grouped them into two groups above and below the average: high Pfirrmann score: 14 and above, and low Pfirrmann score: 13 and below.

### 2.3 Mass spectrometry

From the 326 subjects, we selected a subset of 100 subjects for a serum DIA/SWATH MS to screen for potential biomarkers. There were 50 subjects selected for horizontally and vertically large MCs, half of them with cLBP, half of them with no cLBP, and 50 subjects with noMC, half of them with cLBP, half of them with no cLBP, who were drawn randomly from the larger subset of 326. Serum samples of those participants were analyzed with DIA/SWATH MS to detect all serum proteins. It was performed in the proteomics unit of the Department of Health Sciences and Technology, ETH Zurich, Switzerland: Proteins in serum were digested using the PreOmics iST kit (catalog no. P.O.00027, PreOmics, Planegg/Martinsried, Germany). The procedure protocol is divided into three steps: lysis, digestion, and peptides clean up. For lysis, 2 μL of serum was suspended in 50 μL of lysis buffer and incubated at 95°C for 10 min. Samples were then digested for 3 h at 37°C and peptides were further purified by filter‐based clean up on columns according to the manufacturer's protocol. Samples were analyzed on an Q Exactive™ HF‐X mass spectrometer (Thermo Fisher Scientific, Waltham, MA USA) equipped with an Easy‐nLC 1200 (Thermo Fisher Scientific, Waltham, MA, USA). Peptides were separated on a C18 50 cm EASY‐Spray™ HPLC column (2 μm, 100 Å, 75 μm i.d.(ES903, Thermo Fisher Scientific, Waltham, MA USA)). Peptides were loaded onto an Acclaim™ PepMap™ 100 C18 HPLC TRAP column (3 μm, 75 μm i.d × 70 mm length, Thermo Fisher Scientific, Waltham, MA, USA). Mobile phase A consisted of HPLC‐grade water with 0.1% formic acid, and mobile phase B consisted of HPLC‐grade ACN (80%) with HPLC‐grade water and 0.1% (v/v) formic acid. Peptides were eluted at a flow rate of 200 nL/min using a nonlinear gradient from 4% to 52% mobile phase B in 85 min. For data‐independent acquisition (DIA), DIA isolation windows were set to 15 *m*/*z*, and a mass range of *m*/*z* 400–1210 was covered. A total of 54 DIA scan windows were recorded at a resolution of 30 000 with an AGC target value set to 1 × 10^6^.^17^ Normalized collision energy was at 28%. Full MS spectra were recorded at a resolution of 120 000 with an AGC target set to 3 × 10^6^ and the maximum injection time set to 50 ms. DIA data were analyzed using Spectronaut v14 (Biognosys, Schlieren, Switzerland). MS1 values were used for peptide quantification, peptide quantity was set to mean. Data were filtered using Q‐value with a precursor and a protein Q‐value cut‐off of 0.01 false discovery rate (FDR). Interference correction was performed. Files were searched against an in‐house generated, sample‐specific spectral library. The spectral library was generated with ProteomeDiscoverer 2.4 (Thermo Fisher Scientific, Waltham, MA, USA). Files were searched with MSPepSearch (NIST, USA) and Sequest HT (University of Washington, USA) setting cysteine carbamidomethylation as static, phosphorylation (S, T, Y) oxidation and deamidation (N) as variable modifications. Files were searched against a Uniprot human library (release April 2018) together with standards and common contaminants. FDR was scored using Percolator (target FDR 0.01 as strict, target FDR 0.05 as relaxed).

### 2.4 Cytokine/chemokine measurements

We measured a panel of 46 proteins involved in inflammation, chemotaxis, and angiogenesis with the V‐PLEX Human Biomarker 46‐Plex Kit (catalog no. K15088D, Meso Scale Diagnostics, Rockville, Maryland, USA), in all 326 serum samples, according to the manufacturer's instructions. The panel was selected to contain as much of the markers measured in the studies mentioned above as possible.

### 2.5 Statistics

**FIGURE 2:**
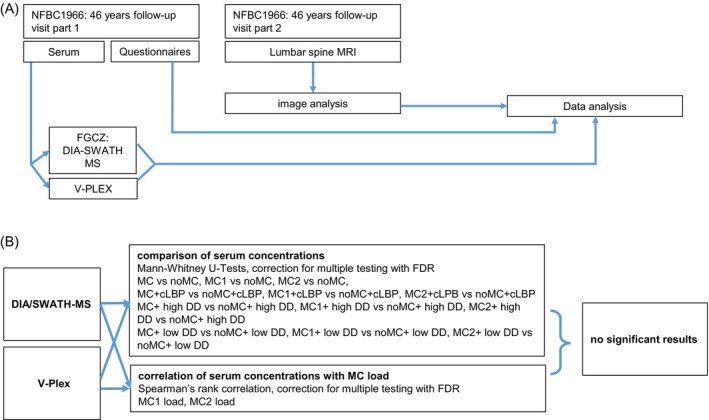
(A) Experimental flow of our study. (B) Diagram of all tests that we performed. There was no significant result. Comparison of serum concentrations: DIA/SWATH‐MS: MC versus noMC: MC *n* = 50; noMC *n* = 50; MC1 versus noMC: MC1 *n* = 50, noMC *n* = 50; MC2 versus noMC: MC2 *n* = 45, noMC *n* = 50; MC + cLBP versus noMC + cLBP: MC + LBP *n* = 23, noMC + cLBP *n* = 24; MC1 + LBP versus noMC + cLBP: MC1 + cLBP *n* = 23, noMC + cLBP *n* = 24; MC2 + cLBP versus noMC+cLBP: MC2 + cLBP *n* = 20, noMC + cLPB *n* = 24; MC + high DD versus noMC + high DD: MC + high DD *n* = 38, noMC + high DD *n* = 11; MC1 + high DD versus noMC + high DD: MC1 + high DD *n* = 38, noMC + high DD *n* = 11; MC2 + high DD versus noMC + high DD: MC2 + high, DD *n* = 34 noMC + high DD *n* = 11; MC + low DD versus noMC + low DD: MC + low DD *n* = 10, noMC + low DD *n* = 37; MC1 + low DD versus noMC + low DD: MC1 + low DD *n* = 10, noMC + low DD *n* = 37; MC2 + low DD versus noMC + low DD: MC2 + low DD *n* = 10, noMC + low DD *n* = 37; V‐Pex: MC versus noMC: MC *n* = 158, noMC *n* = 168; MC1 versus noMC: MC1 *n* = 157, noMC *n* = 168; MC2 versus noMC: MC2 *n* = 145, noMC *n* = 168; MC + cLBP versus noMC + cLBP: MC + LBP *n* = 42, noMC + cLBP *n* = 47; MC1 + LBP versus noMC + cLBP: MC1 + cLBP *n* = 41, noMC + cLBP *n* = 47; MC2 + cLBP versus noMC + cLBP: MC2 + cLBP *n* = 38, noMC + cLPB *n* = 47; MC + high DD versus noMC + high DD: MC + high DD *n* = 30, noMC + high DD *n* = 11; MC1 + high DD versus noMC + high DD: MC1 + high DD *n* = 38, noMC + high DD *n* = 11; MC2 + high DD versus noMC + high DD: MC2 + high, DD *n* = 34 noMC + high DD *n* = 11; MC + low DD versus noMC + low DD: MC + low DD *n* = 30, noMC + low DD *n* = 124; MC1 + low DD versus noMC + low DD: MC1 + low DD *n* = 30, noMC + low DD *n* = 124; MC2 + low DD versus noMC + low DD: MC2 + low DD *n* = 27, noMC + low DD *n* = 124. Correlation of serum concentrations with MC load: DIA/SWATH‐MS: all analyses *n* = 100, V‐Pex: all analyses *n* = 100. High DD = lumbar Pfirrmann sore of 14 or higher, low DD = lumbar Pfirrmann score of 13 or lower.

For statistics, we used the software RStudio version 1.4.1717. To detect differences between groups, we used chi‐square tests, with small groups Fisher's exact test and Spearman's rank correlation. The data from the DIA/SWATH MS were log‐transformed and normalized using quantile normalization. To find a potential biomarker for the presence of MC, we used Mann–Whitney‐U‐tests with correction for multiple comparisons with FDR. To find a potential biomarker for the extent of MC, we searched for associations between serum concentration and MC‐load with Spearman's rank correlation, corrected with FDR. Significance level was α = 0.05 for every test. An overview of all tests we performed is given in (Figure 2).

## 3 RESULTS

### 3.1 Subject characteristics

Basic characteristics of the 326 subjects of the cytokine analysis and the 100 subjects of the proteomics analysis are summarized in Table 1. The mean age of the subjects was 46.3 years at the clinical visit (questionnaires, serum samples) and 47.3 years on the day of MRI. There were more females in the group with noMC (female MC: 90 of 158 (56.9%) vs. female noMC: 115 of 168 (68.4%), *p* = 0.03). There was no significant difference in body mass index (BMI) (average BMI MC: 26 kg/m^2^ vs. noMC: 26.1 kg/m^2^, *p* = 0.99), percentage of overweight (MC: 52.5% vs. noMC 56%, *p* = 0.60) and smoking status (current regular smoker MC: 28 (17.7%) vs. noMC: 21 (12.5%), *p* = 0.74). Comorbidities were distributed equally: diabetes type 1: MC: 3 (1.9%) versus noMC: 2 (1.2%), *p* = 0.68; diabetes type 2: MC: 2 (1.3%) versus noMC: 4 (2.5%), *p* = 0.45; coronary heart disease MC: 4 (2.5%) versus noMC: 0 (0%), *p* = 0.054; emphysema/chronic bronchitis: MC: 4 (2.5%) versus noMC: 1 (0.6%), *p* = 0.21; osteoporosis: MC: 3 (1.9%) versus noMC: 1 (0.6%), *p* = 0.36; any cancer MC: 6 (3.8%) versus noMC: 5 (3%), *p* = 0.76.

**TABLE 1:** Basic characteristics by presence of MC on the MRI.

	*n* = 326					*n* = 100				
	MC	%	noMC	%	*p*	MC	%	noMC	%	*p*
Total	158	48.5	168	51.5		49	49	51	51	
MC1	157	99.4	‐	‐		49	100	‐	‐	
MC2	145	91.8	‐	‐		44	89	‐	‐	
MC3	1	0.6	‐	‐		1	2	‐	‐	
More than one MC type	142	89.9	‐	‐		44	89	‐	‐	
Average Pfirrmann score sum L1‐S1	14.82		12.46		<0.0001	14.79		12.35		<0.0001
Average days between serum sampling and MRI	366.5		367.9		0.89					
Sex: female	90	56.9	115	68.4	0.03	28	57.1	32	62.4	0.43
cLBP	42	26.6	47	28.0	0.82	24	48.9	24	47.1	0.76
Unknown pain status	13	8.2	12	7.1		2	4.1	1	2.0	
Average BMI (kg/m^2^)	26		26.1		0.99	26.9		27.3		0.57
Overweight	83	52.5	94	56.0	0.6	31	63.2	37	72.5	0.24
Unknown BMI	2	1.2	2	1.2		0	0	1	2.0	
Smoking ever regularly	81	51.3	95	56.6	0.25	29	59.2	30	58.8	0.84
Unknown if ever smoking	1	0.6	4	2.3		0	0	2	3.9	
Smoking currently regularly	28	17.7	21	12.5	0.74	8	16.3	6	11.8	0.69
Unknown current smoking status	6	3.8	16	9.5		1	2	7	13.7	
Comorbidities										
Diabetes type 1	3	1.9	2	1.2	0.68	1	2.0	0	0	1.00
Diabetes type 2	2	1.3	4	2.4	0.68	0	0	1	2.0	0.48
Unknown if diabetes	2	1.3	7	4.1		2	4.1	7	13.7	
Coronary heart disease	4	2.5	0	0	0.054	2	4.1	0	0	0.49
Unknown if coronary heart disease	3	1.9	3	1.8		1	2.0	4	7.8	
Emphysema/chronic bronchitis	4	2.5	1	0.6	0.21	1	2.0	0	0	0.49
Unknown if emphysema/chronic bronchitis	8	5.6	11	6.5		2	4.1	6	11.8	
Osteoporosis	3	1.9	1	0.6	0.36	0	0	1	2.0	1.00
Unknown if osteoporosis	3	1.9	3	1.8		1	2.0	3	5.9	
Cancer	6	3.8	5	3	0.76	0	0	1	2.0	1.00
Unknown if cancer	3	1.9	4	2.4		3	6.1	4	11.8	

*Note*: On the left: The sample of 326 participants is shown; on the right: The subset of 100 participants for the mass spectrometry study. Overweight = BMI>25 kg/m^2^. Smoking ever regularly = at least 1 cigarette on at least 5 days/week during at least 1 year; smoking currently regularly = at least 1 cigarette on at least 5 days/week. *p* = *p*‐value of either the chi‐square test, for the comorbidities (due to frequency of comorbidities below 5) Fisher's exact test or, for BMI, average Pfirrmann score and “days between serum sampling and MRI”, Mann–Whitney U‐test comparing MC and noMC.

### 3.2 MRI analysis

The MR scans were performed in all 326 subjects. 158 (48.46%) of the subjects had MC of any type in the lumbar spine. The average number of MC in one individual was 3.25 (range 1–9). Most individuals with MC had several types of MC and mixed types. There were 13 (8.2%) subjects with only MC1, one (0.6%) subject with only MC2 and no subject with only MC3. Mixed type MC were assigned to both MC types, for example, a patient with MC1/2 was assigned to have MC1 and MC2. There was only one subject with MC3, therefore we did not take MC3 into account for further analysis. We listed the summary in Table 1. Most MCs were adjacent to the L5/S1 disc. As a measure for the extent of the MC, we calculated the MC load for each MC type of each individual as the sum of the product of transverse area and height for each MC (Figure 1). In the subjects with MC selected for mass spectrometry, the MC1 load was higher than in the basic population (MC1 load 33.08 vs. 26.45, *p* = 0.0005; MC2 load 30.6 vs. 25.93, *p* = 0.09). In our study neither MC1 load (*p* = 0.61) nor MC2 load (*p* = 0.96) correlated with the presence of cLBP.

Disc degeneration as measured by the Pfirrmann score was more severe in the MC group: MC: 14.82 versus noMC 12.46, *p*<0.0001 with Pfirrmann scores ranging from 10 to 19.

Representative MR images of one subject are presented in Figure 1.

### 3.3 Mass spectrometry

We performed DIA/SWATH‐MS on the subset of 100 serum samples selected for the largest expected effect size. We detected 1075 serum proteins. Of these, 453 passed a stringent 1% FDR filter. Log‐transformed and normalized results are presented in Table S1. We neither found any significant association between any of the 1075 detected serum proteins and the presence of MC, MC1, or MC2, nor a correlation with the MC1 load or MC2 load. There was also no difference if the analysis was restricted to the samples of participants with cLBP or with high (14 or higher) or low (13 or lower) Pfirrmann score (Tables S1 and S3).

### 3.4 Cytokine measurement (V‐plex)

For six of the 46 measured cytokines, more than 50% of the measurements were below detection limit and therefore not considered for further analysis. We neither found any significant association between the serum concentrations of the measured cytokines/chemokine/factors involved in inflammation and angiogenesis and the presence of MC, MC1, or MC2, nor a correlation with the MC1 load or MC2 load. There was also no difference if the analysis was restricted to the samples of participants with cLBP or with high (14 or higher) or low (13 or lower) Pfirrmann score (Tables 2, S2, and S4).

**TABLE 2:** Comparison of the significant results of the studies of Rannou 2007, Boisson 2019, Dudli 2020, Karppinen 2021, Giefsen 2021, Li 2022, and Aboushaala 2024 with our results. ‐ = not measured, n.s. and green = no significant result, n.d. = not detected (DIA/SWATH‐MS) or below detection limit (in all other tests), orange = significant result.

Study	Current study		Rannou 2007	Boisson 2019	Dudli 2020	Karppinen 2021	Giefsen 2021	Li 2022	Aboushaala 2024
Population	Population cohort		Patients hospitalized for cLBP (noMC vs. MC1 and MC2), MC only on 1 level in the lumbar spine	Outpatient patients with cLBP + MC1 versus cLBP + noMC	Outpatient patients with cLBP + noMC vs. MC1, MC2 vs. age and sex‐matched volonteers with noLBP + noMC and MC	Outpatient patients with cLBP + MC1, MC2 or MC1/2 versus volonteers with noLBP + noMC	Outpatient patients with cLBP + MC1, MC2 versus volunteers with noLBP (no MRI)	Population cohort	Patients undergoing spinal fusion or micro‐discectomy surgery with or without MC
*n*	MS: MC n = 50, noMC =50	Cytokine/chemokine panel: MC *n* = 158, noMC *n* = 168	MC1 *n* = 12, MC2 *n* = 2, noMC *n* = 12	MC1 *n* = 13, noMC *n* = 21	MC + cLBP *n* = 19, noMC + cLBP *n* = 19, noLBP + noMC *n* = 14	MC + cLBP *n* = 40, noMC + noLBP *n* = 40	MC1 *n* = 46, MC2 *n* = 37, noMC *n* = 50	Total *n* = 3584	MC = 13, noMC = 18
C‐reactive protein	n.s.	n.s.	Increased in MC1 versus noMC and MC2	‐	‐	n.s.	n.s.	‐	‐
Neo‐epitopes of pro‐collagen type 3	n.d.	‐	‐	‐	Increased in MC1 + cLBP and MC2 + cLBP versus noMC	‐	‐	‐	‐
Neo‐epitopes of pro‐collagen type 4	n.d.	‐	‐	‐	Increased in MC2 versus noMC	‐	‐	‐	‐
Collagen degradation neo‐eiptope type 4	n.d.	‐	‐	‐	Increased in any MC versus no MC	‐	‐	‐	‐
Interleukin‐6	n.d.	n.s.	‐	n.s.	‐	n.s.	Increased in MC1 and MC2 versus controls	‐	n.d.
Interleukin‐8	n.d.	n.s.	‐	n.s.	‐	Decreased in any MC versus no MC	n.s.	‐	n.d.
Tumor necrosis factor‐α	n.d.	n.s.	‐	n.s.	‐	Decreased in any MC versus no MC	n.s.	‐	n.d.
C‐terminal polypeptide‐1	n.d.	‐	‐	‐	‐	Decreased in any MC versus no MC	‐	‐	
Eotaxin‐1/CCL11	n.d.	n.s.	‐	‐	‐	Decreased in any MC versus no MC	n.s.	‐	n.s.
Eotaxin‐3/CCL26	n.d.	n.s.	‐	‐	‐	Decreased in any MC versus no MC	n.s.	‐	n.d.
Macrophage inflammatory protein‐1α/CCL3	n.d.	n.s.	‐	‐	‐	Decreased in any MC versus no MC	n.s.	‐	n.d.
Mast cell protease‐1	n.d.	n.s.	‐	‐	‐	Decreased in any MC versus no MC	‐	‐	‐
Regulated on activation, normal T cell expressed and secreted (RANTES)/CCL5	n.d.	‐	‐	‐	‐	Decreased in any MC versus noMC	‐	‐	Increased in MC vc noMC
TARC /CCL17	n.d.	n.s.	‐	‐	‐	Decreased in any MC versus noMC	n.s.	‐	n.d.
Interleukin‐15	n.d.	n.s.	‐	‐	‐	Decreased in any MC versus noMC	‐	‐	n.d.
Interleukin‐16	n.d.	n.s.	‐	‐	‐	n.s.	Increased in MC1 and MC2 versus controls	‐	n.s.
Tumor necrosis factor‐β	n.d.	n.s.		‐	‐	Decreased in any MC versusno MC	‐	‐	n.d.
Interleukin‐1sRII	n.d.	‐	‐	‐	‐	Increased in any MC versus noMC	‐	‐	‐
Interferon‐γ	n.d.	n.s.	‐	‐	‐	Decreased in any MC versus noMC	n.s.	‐	n.d.
Vascular endothelial growth factor‐C	n.d.	n.s.	‐	‐	‐	Decreased in any MC versus noMC	‐	‐	‐
Vascular endothelial growth factor‐D	n.d.	n.s.	‐	‐	‐	Decreased in any MC versus noMC	‐	‐	‐
Angiopoetin receptor (Tie)‐2	n.d.	n.s.	‐	‐	‐	Decreased in any MC versus noMC	‐	‐	‐
Vascular endothelial growth factor receptor −1	n.d.	n.s.	‐	‐	‐	Decreased in any MC versus no MC	‐	‐	‐
Hepatocyte growth factor	n.d.	n.s.	‐	‐	‐	Increased in any MC versus no MC	‐	‐	n.s.
Intercellular adhesion molecule‐1	n.s.	n.s.	‐	‐	‐	Decreased in any MC versus no MC, neg. correlation between MC and MC1 size and serum concentration	‐	‐	‐
Vascular cell adhesion molecule‐1	n.s.	n.s.	‐	‐	‐	Decreased in any MC versus no MC	‐	‐	‐
Macrophage migration inhibitory factor	n.d.	‐	‐	‐	‐	‐	Increased in MC1 and MC2 versus controls	‐	Decreased in MC versus noMC
CCL20	n.d.	‐	‐	‐	‐	‐	Increased in MC1 versus controls	‐	n.d.
CCL27	n.d.	‐	‐	‐	‐	‐	Increased in MC1 and MC2 versus controls	‐	‐
CX3CL1	n.d.	‐	‐	‐	‐	‐	Increased in MC1 and MC2 versus controls	‐	n.d.
Cholesterol esters in large LDL	‐	‐	‐	‐	‐	‐	‐	Correlated with lumbar MC	‐
Phospholipids in large LDL	‐	‐	‐	‐	‐	‐	‐	Correlated with MC2	‐
Mean diameter for LDL including IDL	‐	‐	‐	‐	‐	‐	‐	Negatively correlated with MC2	‐
Mean diameter for VLDL	‐	‐	‐	‐	‐	‐	‐	Negatively correlated with lumbar MC, indication for causal effect	‐
Phospholipids in chylomicrons and extremely large VLDL	‐	‐	‐	‐	‐	‐	‐	Negatively correlated with MC2	‐

## 4 DISCUSSION

This is the first study to use a completely unbiased proteomics approach on a subset of a population‐based study to find serum biomarkers for MC.

### 4.1 Strengths and limitations

*Strengths*: The NFBC1966 is one of the most meticulously followed population cohorts with extensive documentation and careful handling of biological samples.^14^ MR images were obtained with the same protocol in all subjects, and the same researchers performed the image analysis. Like this, within the study, the inter‐observer variability for the classification of MC was low.^3^

DIA/SWATH‐MS detects proteins with the high sensitivity of shotgun proteomics methods and additionally quantifies the amount of the detected proteins without prior knowledge of the proteins that are to be detected.^12^ It detects a large number of analytes, thus, it is a good screening tool. In addition, it has a highly reproducible and comparable output. Therefore it is a good tool to search for a biomarker.

*Limitations*: We have no information if the subjects, either with MC or with noMC, had additional MC in the cervical or thoracic spine, which could have influence on the serum levels of potential biomarkers. This is a bias that applies to all previously mentioned studies with the exception of Li et al.^11^ MC in the cervical spine is rare, and MC in the thoracic spine is even rarer.^18^ In addition, the cervical vertebral bodies, and thus MCs located there, are small compared to the lumbar vertebral bodies. This reduces their potential influence on serum biomarker levels.

Another potential bias is that the MRIs were performed on average 1 year after the withdrawal of serum samples. To our knowledge, there is no longitudinal study investigating the evolution of MC in the lumbar spine within 1 year, but data from a large study showed that over 3 years, the rate of change of MC (incidence of new lesions, change of type, resolution of the lesion) was low.^19^ This suggests that within 1 year, there was probably no substantial change.

DIA/SWATH‐MS allows measurements of proteins in low abundance. Unfortunately, the detection limit cannot easily be determined. As the V‐plex detected factors that were not detected in the DIA/SWATH‐MS, we conclude that these factors, mainly cytokines and chemokines, were below the detection limit of the DIA/SWATH‐MS. We added the V‐plex measurement to measure cytokines and chemokines, which has the bias of preselection.

### 4.2 Interpretation of conflicting data

Some of the cytokines and chemokines we measured with the V‐Plex were measured in earlier studies^5^, ^6^, ^7^, ^8^, ^10^ but the previous positive findings could not be reproduced in our larger dataset and also not in the other studies mentioned. This can have technical reasons: Different analysis systems can provide different results and serum cytokine concentrations are highly variable and depend on diurnal cycle, food intake, and other acute events. All blood samples of the NFBC1966 were taken in a fasted state, but this was not the case in all studies mentioned above. However, what is probably more important is, that the studies were performed in different populations, we will discuss this below.

### 4.3 Is there a serum biomarker for MC?

We did not find a serum biomarker for MC, MC1, or MC2 in our population‐based study.

It is important to note that the historic classification of MC based on T1 and T2‐weighted images into MC1, MC2, MC3, and mixed types MC1/2 and MC2/3 does not fully depict the pathobiologic reality. Different etiologic and phenotypic subtypes of MC have been described and the degree of inflammation, bone turnover, and fibrosis is probably heterogeneous within one patient, and even within one lesion depending on the status of the adjacent discs and comorbidities.^4^, ^20^, ^21^, ^22^ Different MC subtypes may produce different serum cytokine profiles. Some of these MC subtypes might be associated with pain, and be therefore enriched in cohorts with subjects who presented themselves because of cLBP. This might be a reason that other studies found cytokines or chemokines increased in patients with MC that we did not detect. Even when we restricted our analysis to those subjects with cLBP, our subjects are still different from the subjects presenting themselves for cLBP, or even undergoing surgery for cLBP. Future studies might reproduce serum cytokines/chemokine in these special cohorts, or find serum markers for special MC subtypes.

However, we aimed to find a robust serum biomarker associated with the presence of MC regardless of comorbidities, the situation of the disc, or the different etiologies of MC. For this, we used with DIA/SWATH MS an unbiased yet sensitive approach, and tried to reproduce findings of others with the even more sensitive V‐Plex. The fact that we did not find any potential biomarker for MC with these methods and that the findings of other studies had conflicting results suggests that there is no reliable serum biomarker that fulfills the criteria to be independent of the cohort, independent of comorbidities and independent of etiological subtypes, which could be used for diagnostics. We cannot exclude that such a biomarker exists below the detection limit of the DIA/SWATH MS, but most probably, it is not among the cytokines that were tested before. It could also reflect that MC are a local process confined to the spine, and there is no such blood biomarker. The negative results from this comprehensive MC biomarker screening are important to efficiently allocate research resources in the future to overcome the limitations of the current binary MC classification system and large inter‐rater variability. It is possible that the diagnostic of MC will stay exclusively imaging‐based, which might be defined with new imaging techniques. Therefore, we recommend that future MC biomarker research should focus to improve imaging techniques and data analysis algorithms rather than searching for blood biomarkers.

## CONFLICT OF INTEREST STATEMENT

All authors declare that the research was conducted in the absence of any commercial or financial relationships that could be construed as a potential conflict of interest.

## Supporting information


**Table S1:** Full log‐transformed and normalized data of the DIA/SWATH‐MS in arbitrary units.

**Table S2:** Comparison of the studies on blood biomarkers in MC with all factors tested/detected. MC = Modic changes, cLBP = chronic low back pain, “‐” = not tested, n.s. and green = no significant result, n.d. = for mass spectrometry: not detected, for the other studies: below detection limit. Red: significant results.

**Table S3:** *p*‐values and correlation coefficients of all Mann–Whitney U‐tests and Spearman correlations comparing DIA/SWATH‐mass spectrometry results of MC1 and MC2 to noMC for all detected proteins.

**Table S4:** *p*‐values and correlation coefficients of all Mann–Whitney U‐tests and Spearman correlations comparing V‐plex results of MC1 and MC2 to noMC for all proteins above detection limit.

## Data Availability

NFBC data are available from the University of Oulu, Infrastructure for Population Studies. Permission to use the data can be applied for research purposes via an electronic material request portal. In the use of data, we follow the EU general data protection regulation (679/2016) and the Finnish Data Protection Act. The use of personal data is based on a cohort participant's written informed consent in their latest follow‐up study, which may cause limitations to its use. Please, contact the NFBC project center (nfbcprojectcenter@oulu.fi) and visit the cohort website (www.oulu.fi/nfbc) for more information.
